# The diagnostic role of gut feelings in general practice *A focus group study of the concept and its determinants*

**DOI:** 10.1186/1471-2296-10-17

**Published:** 2009-02-18

**Authors:** Erik Stolper, Marloes van Bokhoven, Paul Houben, Paul Van Royen, Margje van de Wiel, Trudy van der Weijden, Geert Jan Dinant

**Affiliations:** 1 School for Public Health and Primary Care (CAPHRI), Department of General Practice, Maastricht University, Maastricht, The Netherlands; 2 Department of General Practice, University of Antwerp, Antwerp, Belgium; 3 Department of Work and Social Psychology, Maastricht University, Maastricht, The Netherlands

## Abstract

**Background:**

General practitioners sometimes base clinical decisions on gut feelings alone, even though there is little evidence of their diagnostic and prognostic value in daily practice. Research into these aspects and the use of the concept in medical education require a practical and valid description of gut feelings. The goal of our study was therefore to describe the concept of gut feelings in general practice and to identify their main determinants

**Methods:**

Qualitative research including 4 focus group discussions. A heterogeneous sample of 28 GPs. Text analysis of the focus group discussions, using a grounded theory approach.

**Results:**

Gut feelings are familiar to most GPs in the Netherlands and play a substantial role in their everyday routine. The participants distinguished two types of gut feelings, a sense of reassurance and a sense of alarm. In the former case, a GP is sure about prognosis and therapy, although they may not always have a clear diagnosis in mind. A sense of alarm means that a GP has the feeling that something is wrong even though objective arguments are lacking. GPs in the focus groups experienced gut feelings as a compass in situations of uncertainty and the majority of GPs trusted this guide. We identified the main determinants of gut feelings: fitting, alerting and interfering factors, sensation, contextual knowledge, medical education, experience and personality.

**Conclusion:**

The role of gut feelings in general practice has become much clearer, but we need more research into the contributions of individual determinants and into the test properties of gut feelings to make the concept suitable for medical education.

## Background

Most general practitioners (GPs) would recognise that feeling of sudden heightened awareness or alarm, which sometimes emerges during a consultation: "There's something wrong with this patient but I don't know exactly what. I have to do something because a delay can be harmful". It is a non-specific sense of alarm, which may perhaps seem difficult to explain rationally, an almost visceral sense that something serious may be wrong with the patient. Something vague in the patient's story or in the presentation triggers an alert. Sometimes GPs base their clinical decision on this gut feeling alone, even though there is little evidence of the diagnostic value of gut feelings in general practice. Hardly anything can be found about this phenomenon in the medical literature, which mainly focuses on problem-solving and decision-making in diagnostic processes. [1-5] Sometimes it is specified as a useful warning light, which suddenly lights up to announce that there is something unusual. [6] It has also been described as "a wrong feeling as a way to distinguish urgent from non urgent" and "a rough assessment of the situation to identify emergency problems". [7,8] Primary care research into the diagnostic value of signs and symptoms for serious infections in children has identified the physician's feeling that "something is wrong" as most important. [9] A GP's first impression about the seriousness of chest pain is highly reliable. [10] Medical intuition or a 'clinical nose' in diagnostics seems powerful and real, but poorly defined. [11] Despite this, gut feelings were not mentioned in reviews of diagnostic reasoning and medical expertise. [1,2] Our literature search revealed that more is known about the role of gut feelings in neonatal intensive care units and in emergency care settings. [12-14] In this world, full of sophisticated technology, gut feelings appear to be taken seriously because they sometimes alert nurses and doctors to take important action earlier than machines do. [15,16] However, studies about gut feelings and intuition in nursing primarily remain at conceptual and exploratory levels. [17-20]

Although gut feelings thus seem to have a place in the GP's diagnostic process, what is lacking is studies about the validity of this diagnostic instrument. [21] Gut feelings are difficult to examine because they are non-analytical and not easily measurable. But if we were able to find evidence of their positive role in general practice, it could be worth examining the potential for including this aspect of diagnosis and management in medical education. However, research into the value of gut feelings requires an accessible and valid description. In addition, we assumed that a GP's experience and contextual knowledge would be important determinants of the development of gut feelings. In this article we report how we tried to formulate the concept of gut feelings and how we identified the main determinants of such easily recognised but poorly described personal responses to certain clinical situations.

## Methods

### Design

A qualitative approach was chosen because this type of research would enable us to focus on the meaning and significance that GPs attach to gut feelings and opinions about them. We decided to work with focus groups and not with personal interviews since the members of a focus group respond directly to each other, generating more questions about the topic at hand and sharing common experiences while a moderator probes for further explanations. [22,23] A Delphi consensus procedure was not suitable at this stage because of the lack of knowledge about this topic. We opted for purposive sampling to recruit members for the groups, to obtain a representative distribution of factors assumed to be related to the subject, such as experience, gender and urban or rural location of the practice, and to maximize the exploration of different perspectives. We asked the teaching staff at three Departments of General Practice to name GPs in the surrounding areas who were not employed by a university and who might be interested in reflecting on diagnostic thinking. Interested GPs were invited by phone to participate in one of our three planned focus groups. We sent those who agreed to do so written information, without disclosing the exact purpose of the focus groups, so as to avoid bias. For each group of about 7 members, we contacted 10–15 GPs working in the same region. After three focus group sessions had taken place, we concluded that they had included too few inexperienced female physicians. We therefore composed a fourth group, consisting of female GPs who were working part-time and had limited experience as a GP. We developed a scenario in advance, not to steer the discussion but to ensure that all topics relating to our research subject would come up in the discussions. The scenario was adapted after each group because some topics were not clarified satisfactorily (see table 1). For instance, if gender was not spontaneously discussed, it was only included as a topic at the end of the third group meeting.

**Table 1:** some important questions in the scenario

• The aim of this study is to collect information on the way you approach the diagnostic process. When you were training to become a doctor, you learned to diagnose patients using systematic frameworks and questions. In actual practice, however, doctors don't always seem to use such a structured approach, as their gut feelings and practical experience also play an important part. We are especially interested in this non-analytical aspect. What comes to mind when you think about the non-analytical aspects of establishing a diagnosis?
• What happens if your gut feelings start to play a part in the diagnostic process? How do you deal with this? Can you indicate what cues or key symptoms trigger your intuition?
• To what extent do you think this is influenced by professional experience?
• (if this has not yet come up in the discussion) What are your feelings about the 'sense of reassurance versus sense of alarm' distinction? Are these concepts useful in your opinion?
• Can you think of a case in which you had a sense of reassurance which turned out to be unjustified?
• We would like to arrive at a description of such gut feelings (sense of reassurance versus sense of alarm). In your opinion, what elements would definitely have to be included in such a description?
• (after the first group) People in the previous group said that gut feelings are a key element in a doctor's professional behaviour. What do you think of that?
• (after two groups) Do you think the concept of gut feelings (distinguishing between a sense of reassurance and a sense of alarm) can be taught to students?
• (after two groups) In terms of gut feelings, do you think there is a difference between male and female GPs?
• (after two groups) The previous sessions have given us the idea that these gut feelings are more than just feelings, as they also depend on knowledge. What is your opinion about this?

The sessions were chaired by an experienced and independent moderator. The moderator introduced the subject of our research as a discussion about the non-analytical aspects of GPs' diagnostic thinking, [24,25] without mentioning the phrase gut feelings. However, each group spontaneously talked about gut feelings shortly after the group discussions started. The group discussions were tape-recorded and transcribed, and we checked the text. After each meeting there was a debriefing with the moderator and we adapted the scenario to focus on unclear aspects. Data saturation was reached after four group sessions.

### Analysis

Since hardly anything was known about the diagnostic role of gut feelings in general practice, we used the grounded theory approach [26,27] where data are jointly collected, coded and analyzed, while deciding which data belong to which category. We started with an open coding of the transcripts and attached codes to any quote that could be important, in the light of our research questions. Subsequently, we iteratively developed new codes and ideas and compared them with old data. This specific approach is appropriate when studying a previously unresearched phenomenon. It enabled us to construct a theoretical concept, while continuously comparing old data with new ones gathered for this specific purpose. The transcripts were coded by three independent researchers (ES, LvB, TvdW), who reached consensus on the selection of meaningful codes afterwards. In the next phase – known as axial coding – we looked for relations between codes and developed categories and themes to build a grounded theory about gut feelings. Each new step was initiated after agreement in the research group. The analysis was facilitated by the Atlas-ti software program. The text was then reread to reflect on the categories we had developed. Finally, we did a member check by sending the participants a summary of our research findings and incorporating their suggestions for adjustment.

### Ethical approval

Participants were asked to give their informed consent at the start of each focus group session. Since no patients were involved and GPs were only asked about their opinions and perceptions, this research did not fall under the Dutch Medical Research Involving Human Subjects Act (WMO) or the Embryos Act, so that no ethical permission was required.

## Results

### Study population

Four focus group sessions took place, with a total of 28 GPs participating. The characteristics of the GPs met our criteria (see table 2). Two GPs who had accepted the invitation did not turn up, without giving a reason.

**Table 2:** Characteristics of members of focus groups

	N	M	F	Age	Experience < 6 years	Experience > 6 years	Experience, mean no. of years	Urban	Rural	GP trainer	Single- person Pract.	Group Pract.	Part time
F1	6	5	1	45.6	2	4	12.6	0	6	0	3	3	2

F2	6	4	2	49.2	0	6	17.8	4	2	5	4	2	0

F3	9	8	1	50.3	0	9	17.4	5	4	5	3	6	2

**F sub-total**	**21**	**17**	**4**	**48.6**	**2**	**19**	**15.9**	**9**	**12**	**10**	**10**	**11**	**4**

F4	7	0	7	34.7	7	0	4.1	3	4	1	0	7	7

**F total**	**28**	**17**	**11**	**45**	**9**	**19**	**13**	**12**	**16**	**11**	**10**	**18**	**11**

### Describing gut feelings

**Figure 1:**
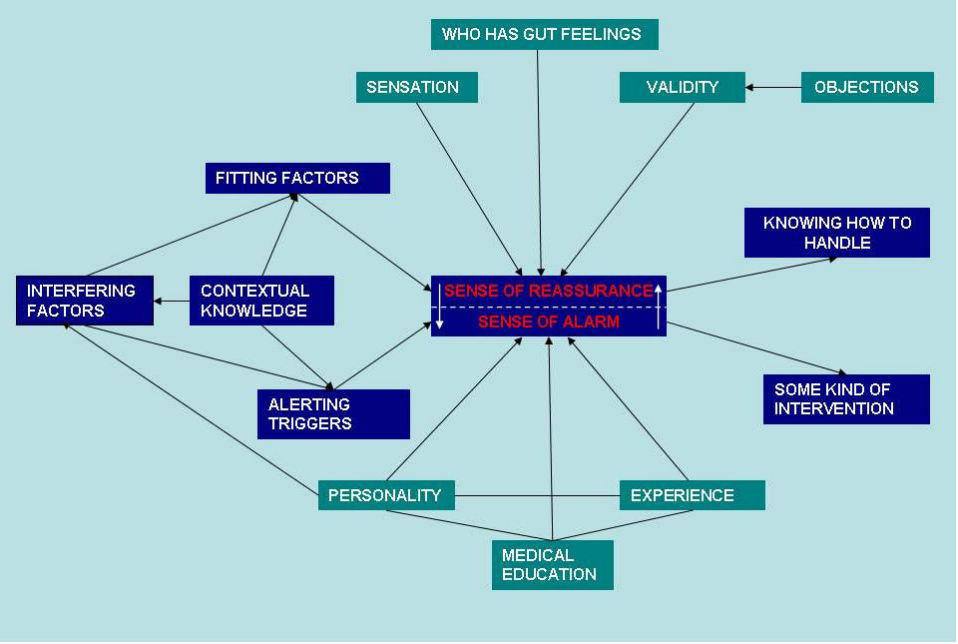
**determinants of gut feelings in general practice**.

Gut feelings were recognized in all focus groups as a phenomenon familiar to most GPs in the Netherlands and playing an important role both in routine practice and during out of hours care. Two types of gut feelings were mentioned by the participants: a sense of alarm and a sense of reassurance. The participants often perceived the sense of alarm as a physical sensation in the abdomen or the heart (a) (see table 3 quotes). Three elements were seen as important in describing a sense of alarm: the feeling that there appears to be something wrong without the doctor having objective arguments, a distrust of the situation because of uncertainty about the prognosis of the complaints and the need for some kind of intervention to prevent serious health problems (b). When they experienced a sense of reassurance, the GPs were sure about the prognosis and therapy, even in the absence of a diagnosis (c). Gut feelings were not related to specific diseases but to the certainty of what a GP had to do. A GP can have a sense of reassurance when he sends one patient with chest pain home but also when he refers another to hospital. GPs were not always conscious of their sense of reassurance at the time they made decisions. It was often identified in retrospect (d). Sometimes a GP experienced a gradually growing sense of alarm, but it might also have a sudden onset, after which it could fade away in the course of the encounter. Several determinants of gut feelings could be distinguished; these are discussed below. Based on our findings we visualized the interrelated determinants in a network (see figure 1: determinants of gut feelings in general practice). The outcome did not differ fundamentally between the groups.

**Table 3:** Quotes

**Defining gut feelings**
a) Where I feel this? Literally in my guts; it's an actual physical sensation, telling me something's wrong. (V1570) I can actually feel my heartbeat start to accelerate. (V1605).
b) It's the feeling that, in spite of all rational arguments and considerations and weighing up all the information you've obtained from history-taking, physical examination and perhaps some additional diagnostics, there's still this underlying feeling of something not fitting in, something being amiss. I can't really grasp it, or put a name on it, and there are all kinds of arguments to say there's nothing wrong, and yet as a GP you still have this sense, which you could call a sense of alarm, of something being not right. (M1444) But to me, this gut feeling means that you're very soon aware whether something is wrong or not. That's the gut feeling. (N591) Because you see a lot of patients with complaints, and with most of them your gut feeling reassures you there's no serious problem. And then suddenly there's one who's not OK and you get this feeling ... a sort of tingling in your spine. (V1599)
c) You've got your diagnosis and it all fits and even if they feel very sick you can say you'll be OK in the morning. So you are backed up by a diagnosis that actually helps you. It all fits, so you're reassured, even though the patient feels very sick. (V2008) But in your everyday practice routine, it's often enough to, say, postpone it or to say it's so recent or things are going OK or whatever, so that means you're working in a grey area, without having an actual diagnosis, but a general sense of what direction to go, or this can wait, or I need to see this patient again. So you're in a grey area: there's as yet no clear diagnosis but you still take a decision. That sort of thing. (M0410)
d) Nine out of ten times, or perhaps even ninety-five out of a hundred times, you're not aware of this sense of reassurance; it's the sense of alarm that you're aware of. (V1215). At a certain moment, it becomes a matter of knowing, this gut feeling of alarm or reassurance, you just know (N0626).
**Fitting or alerting factors**
e) I always think: does this presentation fit in, with the complaints, and with what you find in your examination. Do they form a consistent picture or are there aspects that don't fit in? That make you think wait a minute, this isn't right. And how can I look at it differently? That's when you start to look into it further. (H0501).
f) These people come and, as it were, sing their song. It's usually the same song, but if it changes, that's when you sit up and look at it in a completely different way. (N0385).

**Contextual knowlegde and interfering factors**
g) You also have the frame of reference of the family that a patient comes from, which means you notice when they're different or present in an unusual way or they may say well, this time there's really something wrong with me, or perhaps that's precisely what they do not say, whereas they normally do. So there's something different and that has some significance, in light of what you already know about them. (M0438).
h) When I'm angry like that, my antennae don't work, and that means I'm not being a good doctor to this patient. I'm convinced of that. I really mess up, because my gut feeling no longer works. (N1024).
i) I think my rational considerations, my lists and all that, are much more valid than my initial intuition. I tend to ignore that. (M0747).

**Medical education and experience**
j) It's not what I learned at university; I was taught to work on the basis of lists. (M1296)
k) And I think you can teach an trainee GP this by saying to them wait a minute, stop thinking of numbers and things like that, what about your feelings? What do your feelings tell you? (V2984).
l) Your GP training can provide you with a number of 'handles' that can help you develop this feeling. One of these handles is self-reflection. But it's also a matter of personality: if you're not willing to engage in introspection and self-criticism, you won't easily learn these things. (N2177).
m) The more experienced you are, the more you're able to identify and evaluate the 'noise', and that of course is something I also notice in trainee GPs; they're finding it more difficult, they make less use of the noise than I do. I'm better able to evaluate the importance of the noise and I make better use of it, while they tend to, if they don't understand something they tend to say I don't understand this, so it's probably not important. (M0215).

**Personality**
n) You want to reduce the sense of uncertainty, and personally, my criterion is that I have to be able to sleep quietly at night at any rate; I need to feel I've done the right thing. (M0712). In most cases perhaps you don't know exactly what's going on. But you have a general idea, you have a working diagnosis and I personally don't feel bad about it if that involves a certain degree of uncertainty. (V1314).
o) You receive a whole stream of information through a whole range of channels, and you tend to immediately draw your conclusion from that, but you have to force yourself not to do so, in order to stay at the right level of rationality. Because I think it's a real pitfall. (M272).

**Consequences of a sense of alarm**
p) Those gut feelings of alarm or reassurance, if there's something that makes me worry, that's a feeling that I feel I want do follow up on. They're alarming signals and I need to check them, I need to make sure for myself whether it's something I really need to act upon or whether I can ignore it because it's nothing serious. (N0819). It raises my state of alertness. I tend to literally sit up and start to focus more. (N0412).
q) Those cases in which I think I have a gut feeling that it's OK, but rational arguments say it's not, I always refer those, on rational arguments, to be on the safe side. And cases where rational arguments say it's OK but my gut feelings say there's something wrong, I also refer, based on my gut feeling. (M0754).

**Compass**
r) I had this patient presenting with tightness of the chest, not elicited by exertion, not responding to nitro, nothing in the family history except a younger brother who had some heart complaints at one stage. Apart from that, nothing at all, and yet... He didn't sweat, he seemed very well, and still I had this feeling that I didn't trust the situation. I don't know why... So it turned out he had an inferior wall infarction, and I thought: Yes, I was right! There were no clear indications of an infarction, but I just didn't trust it. And now I won't care if the next four patients I refer turn out to have nothing wrong with them. (M0638)
s) There's a new patient every ten minutes, right, you have to try and understand the problem presented by a patient, you have to ask questions, have the patient undress, do a physical exam, have the patient put on their clothes again, then discuss your findings, explain what you think it is and then make out a prescription and explain about the therapy or try to reassure them before getting ri... err, before getting them to leave, so to say (laughter). And all of that must be done within ten minutes, as you have thirty or thirty-five patients to see that day. So at a certain point you have to, you really need that gut feeling, or you would never get through your surgery, honestly. If you didn't have that gut feeling, you might as well give up tomorrow, I think. This sense of reassurance or alarm, which brings you to your diagnosis, if you haven't got that and always have to rely only on lists and theoretical knowledge, you'd never make it through surgery hour. (H2089).

### Fitting or alerting factors

Many GPs used the phrase: it fits or it does not fit in. They explained this as a process of comparing pictures, that is, comparing the current picture which the overall picture they expected based on what they knew about a patient or about a disease (e). In the case of a sense of reassurance, the current picture was compatible with the known pattern for the patient or for the disease. There was congruity. In the case of a sense of alarm, there was a discrepancy between the pictures. Things did not fit in; something was lacking, or just odd, but the GP did not (or not yet) know exactly what (f). The triggers could be found in a patient's presentation, in the way the patient sat or spoke, or in the way other patients of the same age behaved. It was often a very rapid process: GPs realized these things before they even started reasoning.

### Contextual knowledge and interfering factors

Everything a GP knows about a patient in addition to the presented symptoms and signs, i.e. the contextual knowledge, seemed a very important determinant, because it acted as a frame of reference (g). Interfering factors were mentioned as well: emotions like sympathy, aversions and feelings of guilt from the past could interfere with gut feelings (h). Sometimes GPs reported that they distrusted their gut feelings or disregarded them because of rational considerations (i).

### Medical education and experience

Most GPs in the focus groups believed that gut feelings can be taught, though they are not easily learned. Medical education teaches students to recognize diseases mainly rationally, by selecting and analyzing symptoms and signs step by step, hypothesizing diagnoses and asking supplementary questions: the hypothetic-deductive method (j). But at the same time there is also a diagnostic feeling, a sense of how a patient tells his story or behaves during the consultation, a sense of what is normal for this patient and what is not. GP trainers in the focus groups said that reflection could be a way to develop diagnostic feelings, including gut feelings (k). GP trainers might ask their trainees to stop counting symptoms and numbers and to start listening to what a patient really means, while observing the patient as well as their own feelings (l). Not every sign or symptom would fit in with a diagnosis and the focus group members said that inexperienced GPs tended to ignore these aberrant and individual elements in the flow of information. After several years of experience, however, they used this knowledge to assess the symptoms and signs presented by patients (m). Experience with patients in general practice contributed to the development of gut feelings and made them reliable. GPs developed their own feeling of what is normal or not and familiarized themselves with prior probabilities in their practice; this then became implicit knowledge. In experienced GPs, the whole process of scanning and comparing pictures had become partly automatic. Before applying any logical reasoning, GPs sometimes knew intuitively whether there was something wrong with a patient or whether it was nothing serious.

### Personality

The ability to tolerate uncertainty and to take some risks seems to influence the way physicians handle gut feelings (n). GPs with less self-confidence might not trust their sense of reassurance. Also, they might fear the opinion of colleagues like hospital specialists, which might make them postpone referral to hospital even if they had a sense of alarm. Rational doctors in our focus groups often distrusted gut feelings or had difficulty developing them. Some GPs even regarded gut feelings as a pitfall which they tried to avoid by objective rational diagnostics (o). These GPs pointed out that there is no evidence in the literature for the value of gut feelings.

### Consequences of a sense of alarm

According to the focus group participants, a sense of alarm alerted a physician, and rang an alarm bell. The GP sat up and tried to find objective reasons to support his/her feelings. It thus stimulated the diagnostic process, sometimes resulting in a specific diagnosis (p). But in some cases the sense of alarm remained and the GP had to decide whether to take action or use a policy of watchful waiting (q).

### Compass

GPs are often faced with uncertain situations and gut feelings may act as a compass, which is usually active but not always perceptible. Most of the participants trusted this compass in spite of some misjudgments (r). It steered them through busy office hours and made complex situations manageable (s).

## Discussion

### Main finding

The findings of our focus group sessions show that gut feelings as a diagnostic instrument play a substantial role in general practice and that many GPs rely on it. The participants distinguished two types of gut feeling, a sense of reassurance and a sense of alarm. In the former case a GP is sure about prognosis and therapy, although he may not always have a clear diagnosis in mind. A sense of alarm means that a GP has the feeling that something is wrong even though objective arguments are lacking. He distrusts the situation and is unsure about prognosis and therapy. He feels some kind of intervention is needed to prevent serious health problems. We identified several determinants: fitting, alerting and interfering factors, sensation, contextual knowledge, medical education, experience and personality. Participants denied that gender played any part in the topic. Instead, a GP's rational and emotional characteristics seems to be more important.

### Theory and concept

**Figure 2:**
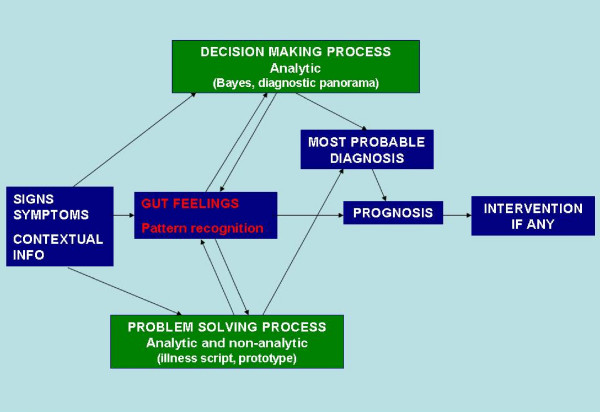
**pathways of GPs' diagnostic reasoning**.

Several years ago, Elstein & Shwarz published a selective review about research into diagnostic reasoning. [2] They distinguished two main schools of thought on the subject. The first is the psychological approach called problem solving, with pattern recognition as an important mechanism and illness script as a model for understanding the knowledge structure. [3,5] The other is the decision-making process, based on probability theory, including parameters such as predictive value, likelihood ratio and diagnostic panorama. [28] We have compiled a diagram to visualize this classification and we suggest that gut feelings should be placed near the centre of the diagram because of their different effects (see figure 2: pathways of GPs' diagnostic reasoning). Gut feelings may stimulate diagnostic reasoning, but when this does not lead to a satisfactory diagnosis, action will be taken. Gut feelings may also bypass explicit reasoning, causing a prompt intervention when a GP considers this necessary. Since determinants like fitting and alerting factors play key roles, pattern recognition seems an important mechanism to explain the gut feelings that arise [1,2,5,29] which is why we have situated gut feelings closer to the problem solving side in our diagram. However, in contrast to what is claimed in the literature on diagnostic reasoning, the pattern of signs and symptoms does not always fit in and does not give rise to a diagnosis, but to a prognosis and/or intervention. The prognosis is then not a specific prediction of the course of a disease but rather a general feeling that action is required. In the case of a compatible, sticking pattern, GPs feel reassured about the prognosis even if they have as yet no clear diagnosis. We suppose that gut feelings act as a diagnostic instrument that is always active, even though doctors are not always aware of it.

Our description of gut feelings is composed of elements mentioned by the GPs in our focus groups. To ensure that our concept is complete and operational, consensus may have to be achieved by means of a Delphi procedure with experts.

### Trustworthiness

Three independent researchers studied the texts of the focus groups and coded them individually to increase the trustworthiness of the data analysis. Afterwards, it appeared that 90% of the codes were similar and consensus on disagreements was easily reached. A different group of researchers might have picked slightly different quotations and would probably have coded somewhat differently, but the element of the researchers being part of the process is characteristic of qualitative research analysis, and the possible bias seems small.

### Variation

Although we now know the essential elements making up the concept of gut feelings, we do not yet know how much they contribute and interact in real practice. GPs vary in the degree to which they rely on gut feelings. Part of this variation may be explained by differences in medical education, while another factor may be the level of experience. How many years of experience in medical practice are necessary to develop and accurately use gut feelings? In our focus groups even GPs with limited experience reported having gut feelings and using them. According to some members of our focus groups, differences in personality play an important role.

The significance of gut feelings can be affected by the position of GPs within their national healthcare system. In the Netherlands, GPs do not work in hospitals but instead act as gatekeepers. Patients consult their GPs, who weigh the presented signs and symptoms against the background of their contextual knowledge, mostly without X-ray or lab results. [30] Dutch GPs, like those in several other countries, follow their patients, often over many years, and thus know much about their history and background. [31,32] It seems interesting to study the significance of gut feelings in other health care systems.

## Conclusion

Most GPs were positive about the significance of gut feelings in general practice and about possibilities to integrate gut feelings in medical education. Although the role of gut feelings in general practice has become much clearer, further research into this complex topic is needed to unravel each determinant's contribution, to examine the accuracy of gut feelings and to make this concept suitable, if possible, for inclusion in medical school curricula. A Delphi consensus procedure may consolidate the elements of the concept of gut feelings and make it operational. We intend to explain gut feelings in the light of current psychological theories and to develop appropriate designs to further study this fascinating phenomenon.

## Competing interests

The authors declare that they have no competing interests.

## Authors' contributions

All authors have made substantial contributions to conception and design. ES organised and attended all the focus groups, executed the analysis and wrote the paper. PvR, MvdW, PH and GJD participated in the design of the study and the development of the focus group scenario. LvB en TvdW were involved in analyses of the data. All authors have discussed the interpretation of the data, and have been involved in drafting the manuscript and have given final approval of the version to be published.

## Pre-publication history

The pre-publication history for this paper can be accessed here:



## References

[B1] Norman GR, Eva K, Brooks LR, Hamstra S, Ericsson KA, Charness N, Feltovich PJ, Hoffman RR (2006). Expertise in Medicine and Surgery. The Cambridge Handbook of Expertise and Expert Performance.

[B2] Elstein AS, Schwarz A (2002). Clinical problem solving and diagnostic decision making: a selective review of the cognitive literature. BMJ.

[B3] Charlin B, Tardif J, Boshuizen HP (2000). Scripts and medical diagnostic knowledge: theory and applications for clinical reasoning instruction and research. Acad Med.

[B4] Elstein AS, Shulman L, Sprafka S (1978). Medical Problem Solving: an analysis of clinical reasoning.

[B5] Schmidt HG, Norman GR, Boshuizen HP (1990). A cognitive perspective on medical expertise: theory and implication. Acad Med.

[B6] Hull F, Sheldon M, Brooke J, Rector A (1985). The consultation process. Decision Making in General Practice.

[B7] Andre M, Borgquist L, Foldevi M, Molstad S (2002). Asking for 'rules of thumb': a way to discover tacit knowledge in general practice. Fam Pract.

[B8] Boreham NC (1994). The dangerous practice of thinking. Med Educ.

[B9] Bruel A Van den (2006). The value of signs and symptoms for the diagnosis of serious infections in children in primary care.

[B10] Buntinx F, Truyen J, Embrechts P, Moreel G, Peeters R (1991). Chest pain: an evaluation of the initial diagnosis made by 25 Flemish general practitioners. Fam Pract.

[B11] Barraclough K (2006). Medical intuition. BMJ.

[B12] Grossman SC, Wheeler K (1997). Predicting patients' deterioration and recovery. Clin Nurs Res.

[B13] Hams SP (2000). A gut feeling? Intuition and critical care nursing. Intensive Crit Care Nurs.

[B14] Nordberg M (1996). Just a gut feeling. Emerg Med Serv.

[B15] Pyles SH, Stern PN (1983). Discovery of Nursing Gestalt in critical care nursing: the importance of the Gray Gorilla syndrome. Image J Nurs Sch.

[B16] Eraut M (2005). Expert and expertise: meanings and perspectives. Learning in Health and Social Care.

[B17] Rew L, Barrow EM (2007). State of the science: intuition in nursing, a generation of studying the phenomenon. ANS Adv Nurs Sci.

[B18] Hamm RM, Dowie J, Elstein A (1988). Clinical intuition and clinical analysis: Expertise and the Cognitive Continuum. Professional judgement A reader in clinical decision making.

[B19] Dreyfus HL, Dreyfus SE (1986). Mind over machine: The power of human intuition and expertise in the era of the computer.

[B20] Benner P, Tanner C (1987). Clinical judgment: how expert nurses use intuition. Am J Nurs.

[B21] Greenhalgh T (2002). Intuition and evidence-uneasy bedfellows?. Br J Gen Pract.

[B22] Pope C, Van Royen P, Baker R (2002). Qualitative methods in research on healthcare quality. Qual Saf Health Care.

[B23] Vermeire E, Van Royen P, Griffiths F, Coenen S, Peremans L, Hendrickx K (2002). The critical appraisal of focus group research articles. Eur J of Gen Pract.

[B24] Eva KW (2004). What every teacher needs to know about clinical reasoning. Med Educ.

[B25] Bowen JL (2006). Educational strategies to promote clinical diagnostic reasoning. N Engl J Med.

[B26] Glaser B, Strauss A (1967). The Discovery of Grounded Theory.

[B27] Strauss A, Corbin J (1998). Basics of qualitative research: techniques and procedures for developing grounded theory.

[B28] Van Puymbroeck H, Remmen R, Denekens J, Scherpbier A, Bisoffi Z, Ende J Van den (2003). Teaching problem solving and decision making in undergraduate medical education: an instructional strategy. Med Teach.

[B29] Hani MA, Keller H, Vandenesch J, Sonnichsen AC, Griffiths F, Donner-Banzhoff N (2007). Different from what the textbooks say: how GPs diagnose coronary heart disease. Fam Pract.

[B30] Baerheim A (2001). The diagnostic process in general practice: has it a two-phase structure?. Fam Pract.

[B31] Hjortdahl P (1992). The influence of general practitioners' knowledge about their patients on the clinical decision-making process. Scand J Prim Health Care.

[B32] Hjortdahl P, Jones R, Britten N, Culpepper L, Gass DA, Grol R, Mant D (2004). Continuity of care. Oxford Textbook of Primary Medical Care Principles and Concepts.

